# QTLminer: identifying genes regulating quantitative traits

**DOI:** 10.1186/1471-2105-11-516

**Published:** 2010-10-15

**Authors:** Rudi Alberts, Klaus Schughart

**Affiliations:** 1 Department of Infection Genetics, Helmholtz Centre for Infection Research and University of Veterinary Medicine Hannover, Inhoffenstrasse 7, 38104 Braunschweig, Germany

## Abstract

**Background:**

Quantitative trait locus (QTL) mapping identifies genomic regions that likely contain genes regulating a quantitative trait. However, QTL regions may encompass tens to hundreds of genes. To find the most promising candidate genes that regulate the trait, the biologist typically collects information from multiple resources about the genes in the QTL interval. This process is very laborious and time consuming.

**Results:**

QTLminer is a bioinformatics tool that automatically performs QTL region analysis. It is available in GeneNetwork and it integrates information such as gene annotation, gene expression and sequence polymorphisms for all the genes within a given genomic interval.

**Conclusions:**

QTLminer substantially speeds up discovery of the most promising candidate genes within a QTL region.

## Background

Quantitative trait locus (QTL) mapping is a powerful method to identify genes regulating complex traits. By combining molecular marker data of genetically related individuals with phenotypic trait values, genomic QTLs are identified that likely contain genetic regulators of the trait. This strategy has both been applied to 'classical' traits like body weight, blood pressure or disease susceptibility, as well as to traits measured using high-throughput technologies: mRNA abundances measured by microarrays [1,2], and protein or metabolite abundances measured by mass spectrometry [3,4]. QTLs generally span a genomic region containing tens to hundreds of genes. Identification of the most promising regulating genes within QTL intervals, which can then be functionally tested, still remains a major challenge. QTLminer has been implemented in the GeneNetwork [5], a large resource with genotypes, phenotypes and gene expression profiles for multiple organisms and genetic reference populations. It automatically analyses a QTL region and integrates information about the candidate genes, so that the best candidate genes can be quickly identified.

## Implementation

QTLminer was implemented in Python as part of the GeneNetwork [5].

## Results and Discussion

QTLminer takes a QTL interval as input, which is defined by the chromosome and the start and end positions in megabases. The program automatically generates a list of genes within the interval and retrieves additional information for each gene. The first part comprises annotation data such as gene name, description, genomic position, Gene Ontology (GO) terms and KEGG pathways in which the gene is implicated. Next, the amount of non-synonymous single nucleotide polymorphisms (nsSNPs) within the gene is displayed. nsSNPs result in amino acid changes in the corresponding protein. These changes may modify its structure and may thus be causative for the phenotypic differences which were mapped. Furthermore, the user can select three GeneNetwork expression data sets. For each of the data sets, gene expression and information about *cis*-regulation will be added. In this way, the user can see whether candidate genes are expressed in the tissue under study. Genes that are only expressed in the tissue of interest and not in others might even be better candidates. The user should however be aware that high expression is not always required, *i.e*. lowly expressed genes may also have a strong influence on traits. In addition, information about *cis*-regulation is added. A gene is *cis*-regulated if its expression maps close to its own genomic position. Any *cis*-regulated gene in the QTL region is a good candidate to regulate the trait, since *cis*-regulation indicates a difference in gene expression levels, which may regulate the trait. Alberts et al. [6] have shown that 'ghost' *cis*-eQTLs (expression QTLs) can be detected if there are SNPs or other sequence variants in the probe regions that cause a difference in hybridization signal. These cases might also be interesting since the sequence variants in the probe regions might result in changes in the protein structure which can regulate the trait.

To demonstrate the utility of the program, we took a QTL hotspot on mouse distal chromosome 1 as example. This region, also called Qrr1, contains many QTLs that control neural and behavioral phenotypes, including motor behavior, escape latency, emotionality, and seizure susceptibility (Szs1) [7]. Mozhui et al. have further investigated this region and revealed a highly complex gene expression regulatory interval in Qrr1, composed of multiple loci modulating the expression of functionally cognate sets of genes. In the distal part of Qrr1, they have identified the gene *Fmn2 *as a strong candidate. To re-analyze this interval using QTLminer, open a web browser and go to http://genenetwork.helmholtz-hzi.de and click 'Search' and 'QTLminer'. In the form that appears, choose Chromosome 1, view from 173 Mb to 177 Mb. Select two mouse strains for which nsSNPs should be analyzed. Choose three GeneNetwork data sets and click 'Analyze QTL interval'. Three hippocampus data sets in BXD, CXB and LXS mice are chosen by default.

**Figure 1:**
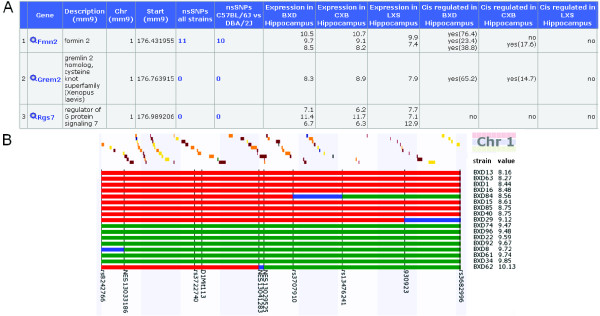
**QTLminer results and haplotype visualization**. A) Part of the results returned by QTLminer. The genes in the QTL interval and their descriptions and positions are presented. Next, the amount of non-synonymous SNPs within all available mouse strains is given, and the amount of nsSNPs between two chosen strains, in this case C57BL/6J and DBA/2J. The expression columns display the mean expression values within three GeneNetwork data sets. Note that multiple values per gene are given when the gene is measured by multiple probes. The next columns show *cis*-regulation. If a gene is *cis*-regulated, the LRS value (likely ratio statistic) that indicates the significance of the *cis *QTL is also given. B) Haplotype visualization of BXD mice for the Qrr1 region. A red (green) line indicates that the mouse carries the C57BL/6J (DBA/2J) genotype. The vertical lines are existing genotypic data and colored lines in between are inferred genotypes based on the mapping of surrounding markers. Blue lines indicate a region containing a recombination break point. Strains are ordered according to trait value. Genes in the interval are displayed at the top (colored bars).

Figure 1A displays the results of QTLminer. It shows the genes within the Qrr1 interval, as well as their descriptions and positions. To obtain more information about the function of the genes, Gene Ontology terms and all KEGG pathways in which each gene occurs are displayed (excluded from the figure). Next, the amounts of (strain specific) nsSNPs are shown. Clicking this number links to GeneNetwork's SNP browser where detailed information about the SNPs can be searched. In the expression and *cis *columns, one can directly see which genes within the QTL interval are expressed and *cis*-regulated, and also compare expression and *cis*-regulation to other tissues (data sets). The user should, however, be aware that often gene expression values are measured in whole organs. These values might not reflect the expression values in a specific cell type of interest, especially if the amount of this cell type is only a small fraction of the total number of cells in the organ. In the Qrr1 interval, over 100 genes were found. Indeed the gene *Fmn2 *shows up as a very good candidate, because it exhibits a high expression value, strong *cis*-regulation and contains nsSNPs between the parental strains C57BL/6J and DBA/2J. To help the user to interpret the results, we added a score to each gene and the possibility to sort the genes according to either position or score. The score ranges between 0 and 4 and increases by steps of one unit if 1) the gene has an expression value greater than 8 in the first data set; 2) the gene is *cis*-regulated in the first data set; 3) the genes contains non-synonymous SNPs; 4) the gene contains indels. It should be noted that these scores are just helping the biologist user to sort the list and to get the most interesting candidates at the top of the results table. The user should still study gene descriptions, pathways and other information of all genes to get the best candidates. A gene with a score of 1 which is known to be involved in the biological process under study might be a much better candidate than a gene with unknown function and a higher score.

An additional feature of QTLminer is the visualization of the haplotypes within a QTL region. In the haplotype plot (Figure 1B), individuals are sorted according to their quantitative trait value, and their haplotypes are indicated by colors. The gene *Kcnj9 *is one of the genes with a strong trans eQTL in the Qrr1 interval in hippocampus. To obtain BXD haplotypes for this gene, search the BXD hippocampus data set in GeneNetwork for *Kcnj9*, click probeset 1450712 at, click the button 'Interval Mapping' and fill in Chromosome 1, 173 until 177 Mb. Select 'Haplotype Analyst' and click 'Remap'. It is immediately obvious that mice with a C57BL/6J (red) allele at the QTL location have a low trait value, whereas mice with a DBA/2J (green) allele have a high trait value. This visualization may be used for fine mapping QTLs. Suppose that a QTL separates two groups of individuals with low and high trait value for most individuals, but some individuals (which were not yet studied) have a recombination within the interval. Then these individuals may be used for further genotyping and in this way, a QTL region can be further narrowed down.

## Conclusions

QTLminer automatically integrates gene annotation, Gene Ontology terms, KEGG pathway information, gene expression and *cis*-regulation data for all genes within a QTL interval. With only a few mouse clicks on the GeneNetwork website, the most promising candidate genes within a given QTL region are quickly highlighted.

## Availability and requirements

**Project name**: QTLminer

**Project home page**: http://genenetwork.helmholtz-hzi.de (click Search - QTLminer)

**Operating system(s)**: Platform independent

**Programming language**: Python

**Other requirements**: none

**License**: none

**Any restrictions to use by non-academics**: none

## Authors' contributions

RA conceived the study, wrote the manuscript and implemented the program. KS conceived the study and wrote the manuscript. All authors read and approved the final manuscript.

## References

[B1] Brem RB Yvert G Clinton R Kruglyak L Genetic dissection of transcriptional regulation in budding yeast Science 2002 296 752 755 10.1126/science.1069516 11923494

[B2] Bystrykh L Weersing E Dontje B Sutton S Pletcher MT Wiltshire T Su AI Vellenga E Wang J Manly KF Lu L Chesler EJ Alberts R Jansen RC Williams RW Cooke MP de Haan G Uncovering regulatory pathways that affect hematopoietic stem cell function using 'genetical genomics' Nat Genet 2005 37 225 232 10.1038/ng1497 15711547

[B3] Foss EJ Radulovic D Shaffer SA Ruderfer DM Bedalov A Goodlett DR Kruglyak L Genetic basis of proteome variation in yeast Nat Genet 2007 39 1369 1375 10.1038/ng.2007.22 17952072

[B4] Keurentjes JJ Fu J de Vos CH Lommen A Hall RD Bino RJ van der Plas LH Jansen RC Vreugdenhil D Koornneef M The genetics of plant metabolism Nat Genet 2006 38 842 849 10.1038/ng1815 16751770

[B5] GeneNetwork http://genenetwork.helmholtz-hzi.de

[B6] Alberts R Terpstra P Li Y Breitling R Nap JP Jansen RC Sequence polymorphisms cause many false cis eQTLs PLoS ONE 2007 2 e622 10.1371/journal.pone.0000622 17637838 PMC1906859

[B7] Mozhui K Ciobanu DC Schikorski T Wang X Lu L Williams RW Dissection of a QTL hotspot on mouse distal chromosome 1 that modulates neurobehavioral phenotypes and gene expression PLoS Genet 2008 4 e1000260 10.1371/journal.pgen.1000260 19008955 PMC2577893

